# Opium

**Published:** 1861-10

**Authors:** 


### Opium.

*Tests for Opium.*—Where opium cannot be detected by the smell, Mr. Taylor prefers the sesquichloride of iron as a test, discovering as it does meconic acid in one hundred and sixtieth of a gr. of opium. It might be supposed, says he, that if, on adding strong nitric acid to a portion of the liquid, a bright red color resulted; this would be a sufficient indication of the presence of morphia, and therefore of opium; but a serious mistake might be committed in such a case, unless the operator had previously employed the iron test, and determined the presence of meconic acid in the liquid. It is worthy of remark, that the nitric acid test, while it destroys the color given by the meconate of iron (a dark red,) will bring out when added to excess to the same portion of liquid, the peculiar bright amber-red tint which it is known to give in a solution of morphia. The tests for meconic acid and morphia may be thus applied to one quantity of liquid.

Mr. Taylor found that nitric acid detected one-fifteenth gr. of muriate of morphia, diluted in 300 parts by weight of water; sesquichlor. iron detected the one-eleventh gr. in 231 parts of water; and iodic acid the one hundredth grain in 1300 parts of water. Thus, *iodic acid* is by far the most delicate test, discovering as it does morphia in less than one-fifth of a grain of opium; but it is also the one most open to fallacy, and cannot be employed in colored organic liquids, containing these small quantities. Practically, its utility is far less than would be anticipated from the result of experiments upon the pure salts of morphia. Other experiments were performed for the purpose of ascertaining how small a quantity of *meconic acid* need be present in a fluid to admit of its separation by acetate of lead, and subsequent identification by sesquichlor. of iron. No precipitate of meconate of lead occurred when the proportion of meconic acid was less than one-forty-eight gr., *i. e.*, about 0.34 gr. common opium. Unless, indeed, soluble matter of several grains of opium exists in the liquid for analysis, it will be difficult to obtain meconic acid and morphia separately. The *iron test* for *meconic acid* is far more delicate than any of the tests for morphia, and is much less liable to be interfered with. The one-fiftieth gr. or smallest visible portion of solid meconic acid is easily detected when free, while in solution, in a small quantity of liquid—even one-five-hun. dredth gr. may be discovered. Thus, the presence of this acid may be determined in a liquid from a much smaller quantity than would suffice to form a separable precipitate of meconate of lead; for, while for this latter one-third of a grain of opium is required, less than one-hundredth of a grain suffices for the exhibition of the acid by the direct application of the iron test. The procural of the precipitate of meconate of lead does not increase the certainty of the iron test, but merely enables us to obtain the meconic acid in a concentrated and solid form. *Part* xi. *p.* 100.

*Opium.*—In reference to the employment of opium generally, the constipation which it causes renders it obnoxious to some constitutions. If this (as is believed) arises from an arrest of the biliary secretions, the combination with mercury, rhubarb, or colchicum, will obviate it. In cases where any of these are not admissible, the ox-gall comes to our aid; and whether it is by directly stimulating the secretion of bile, or acting as a substitute for it, there can be no question of its being able to counteract the constipating tendency of opium. But this power of causing constipation becomes available in the treatment of a very formidable disease—namely, in peritonitis from perforation of a portion of the intestinal canal; and as our object here is to arrest the action of the intestines, to enable the opening to be sealed up with organized lymph, it is evident, that to effect this object, the *uncombined* use of opium can only be relied on. Should the aid of mercury be required to combat the consequences of the inflammation, its administration must be postponed to an after-period. *Part* xv., *p.* 87.

*Muriate of Opium.*—The following is Dr. Nichol's formula: Take of the best powdered opium, ℥j.; muriatic acid, ℥j.; distilled water, ℥xx. Mix. Shake this mixture very frequently every day, during fourteen days; then strain and filter. The does is from twenty to forty drops, according to circumstances.

Dr. N. says: I have found, by experience, that this is the best anodyne I am acquainted with. I may mention that I prepared solutions of opium with ascetic, nitric, sulphuric, citric, tartaric, and muriatic acids, and also prescribed them, but the muriatic solution was vastly superior to any one in every respect. All of them produced headache, with the exception of the muriatic. I prefer muriate of opium to the tincture, wine, or powder of opium, and also to the muriate and acetate of morphia; in fact, to any other preparation of opium. It never makes my head ache, but all other preparations do. *Part* xvii., *p.* 303.

*Opium—Nausea from.*—To counteract the nausea from opium, combine with it 30 minims of dilute sulphuric acid.

The nausea from hydrochlorate of morphia is best controlled by dilute hydrochlorate acid. *Part* xxix, *p.* 326.

*Opium in the Treatment of Chronic Ulceration of the Legs, etc.*—Mr. Skey of St. Bartholomew's Hospital, says: I venture to attribute to this remarkable drug the property of promoting the formation of healthy granulations on a surface that, notwithstanding all the previous appliances of surgery, is yet flat, pale, and ungranulating. Now, there is no example of the power of opium to effect this object more conclusive, or in which there is more work to be done, than that form of disease of which I am speaking—which consists of a gap formed on the surface of the body, of greater or less depth and diameter, and in which there exists not even a trace of a curative action—and yet the object is accomplished by means of this agent, and often with remarkable celerity. We call opium a stimulant and a sedative. As a stimulant, it is not very often employed in practice: while its properties, as a sedative, are well known, and are in daily requisition. Its property of mitigating pain, and of promoting sleep, is that for which it is almost exclusively employed, and so completely is its action associated with this sedative principle, that its occasional influence as a stimulant is almost entirely lost sight of, and the stimulating property has merged in the supposed sedative.

I believe that its sedative properties have little concern with the result. In truth, opium is a most valuable stimulant of the vital powers, and whether its action originate with the centre or the periphery of the circulation, whether primarily on the heart or on the capillary vessels, I do not pretend to know; but there is no drug, simple or composite, known to our pharmacologists that possesses an equal power with opium, of giving energy to the capillary system of arteries, of promoting animal warmth, and thus maintaining an equable balance of the circulation throughout the body. To maintain the balance of the circulation! How much of meaning is attached to these words! How many affections of the bodily frame may not be brought within the range of this definition! Take the common chilblain; what is it but a local congestion of blood, caused by defective capillary power?—there is no better remedy than opium; cold feet, as characterizing a person or a constitution, equally relieved; senile gangrene, the result of arrest of the capillary circulation, or its apparent opposite, local hyperemia—these diseases, one and all, manifest a loss of local power, a failure in the balance of the circulation. The term "inflammation," a word formerly in the mouths of our professional brethren on all occasions, is now limited in its application, and should be yet more limited, and I believe, in a yet more advanced state of medical science will be restricted to an actually rare condition of the system. The influence of opium in such conditions, is that of promoting a genial warmth over the system, a glow exactly resembling, and in fact identical with, that produced by the reaction on the system which is caused by the cold-bath. *It is local health*, and the sensation is most agreeable. The benefit derived from opium, when administered for the purpose of arresting inflammatory action of the vessels, admits, I think, of much doubt, and should be resorted to with some hesitation as a remedial agent, though I am quite persuaded that the evil of its administration is greatly overrated. But who will profess ignorance in these days of the inestimable value of this agent when resorted to immediately after an attack of inflammation has been subdued by a local or general bleeding? Here we can imagine that, the activity of the disease being checked, the diffusing influence of opium on the circulation may act as a simple derivative, operating on the vessels at the moment they are not indisposed to yield up their blood, and to which indeed they are compelled by the diffusive power of the general stimulus.

Many years ago, and before the introduction of railway traveling, I was summoned late one afternoon to see a patient some eighty miles from London. I traveled outside the mail. This occurred in the month of December, and the night was extremely cold. By some mistake I omitted to bring my great-coat; and, for the first hour, I suffered a good deal. On reaching a town at some ten miles' distance from London, I took the opportunity, while changing horses, to run across the street to a druggist's shop, where I ordered a draught, containing twenty-five drops of tincture of opium. I believe I was the only person outside the coach that night who did not suffer the slightest sensation from cold.

But it will be urged by many, who have experimented on, and who have observed less than I have done, the medical properties of opium, the infinite importance of studying the reactive effects of this daily poison, and they would inquire into the condition of a person so treated on the following day. You may be assured that it amounts to *nil.* You will, I am sure, readily understand what I mean when I say the cold and the opium mutually balanced and mutually neutralized each other. There could be no reaction, because the influence of the depressing agent—viz., the cold, rather than otherwise, exceeded in duration that of the stimulant. If the period of prostration were brief, and limited to one or two hours, the argument might hold; but it is but a sorry objection to be urged after all.

I wish I could impress on the minds of the medical authorities in the Crimea the real benefit that might be derived to our troops, beaten down by intense cold and suffering in its various forms, from the judicious administration of opium. If twenty-five or thirty drops of tincture of opium, in addition to his ordinary quantum of rum, were administered to each soldier whose nightly services are required in the trenches or on guard, you would hear little complaint of cold for that night, neither would it produce the smallest tendency to sleep.

If cold beget suffering, opium is the antidote to that suffering, and the one will assuredly neutralize the other.

Notwithstanding the prejudices and the bigotry that have long beset the public mind on this subject, and from which our profession is not totally exempt, there is no comparison to be drawn between the practice of dram-drinking and the excessive indulgence in the use of opium.

The man who indulges in spirituous liquors makes daily inroads on his digestive powers not less than on his brain. His appetite is destroyed, and the pabulum for his blood is withheld from his circulation. He is stamped for life, and his perfect health is irrecoverable. The influence of opium, when taken as a means of indulgence, though deleterious, is not permanently injurious. It exercises no serious influence on his digestion or on his cerebral organs, and, the practice once controlled, leaves him in a condition to regain, without difficulty, the fullest vigor of both bodily and mental health.

I have related to you the particulars of several cases of chronic ucler in which recovery was attributable to the medicinal properties of opium, and almost to opium alone. The character of these ulcers strongly marks the inactivity of their nature, and hence the class of society to which they belong. They are marked by a flat base, which indicates, by its pale, flabby uniformity of surface, that no reparatory action has approached it. It is often surrounded by a thick, high ridge of lymph, covered by unhealthy integuments. The depth of the ulcer, which may be seven or eight lines, is caused partly by the ridge, and partly by the excavation of the ulcer below the natural level of the healthy integuments. So long as this ridge exists, although granulations may form, and will form, from the date of the employment of the opium, yet cicatrization will never complete the process of cure unless the wound or ridge be absorbed. Now the action of opium is not alone exhibited in the development of healthy granulations, but in the entire complement of such actions as are required by the sore, viz., the formation of new material, and the absorption of the old.

The influence of the stimulant is exhibited therefore not on one particular function. It does not merely promote secretion, but it stimulates the healthy vital actions in their entirety—viz., secretion, organization, and absorption contemporaneously; the granulations are secreted and organized, while the circumvallation of unsound material, the product of years of growth, is gradually absorbed and reduced to the level of the surrounding integument; for the removal of this wall is quite as indispensable to the ultimate result as the obliteration of the cavity by granulation. Without the two surfaces be brought to the same level, the process of cicatrization, or skinning over, will never be perfected.

If, therefore, we find that a disease like that I have described, and which exhibits so palpably a dormant condition of the remote capillaries, is amenable to this form of stimulant, which can only accomplish the cure by the substitution of healthy for morbid actions, why should we restrict its employment to this class of diseases? Why, as I have elsewhere inquired, may we not experiment with success on any local disease dependent on the same cause—viz., an inert condition of the remote vascular system?

In claiming for Opium the merit of rousing into healthy action the dormant capillary system, to the end of accomplishing the permanent cure of ulcer of the legs in old persons, I by no means wish you to infer that I consider all other modes of treatment unworthy of trial. Indeed, I attach great value to that recommended by Mr. Baynton, of Bristol, and others; but, having tested their value, I have no hesitation in pronouncing that which I have recommended, so far as I am competent to judge, as by far the most certain and efficacious.—*Braithwaite's Epitome, Part* xxxi., *p.* 192.

